# Remote Electroencephalography Monitoring of Epilepsy in Adults: Protocol for a Scoping Review

**DOI:** 10.2196/33812

**Published:** 2022-02-25

**Authors:** Madison Milne-Ives, Rohit Shankar, Brendan McLean, Jonas Duun-Henriksen, Lykke Blaabjerg, Edward Meinert

**Affiliations:** 1 Centre for Health Technology University of Plymouth Plymouth United Kingdom; 2 Peninsula Medical School Faculty of Health University of Plymouth Plymouth United Kingdom; 3 Cornwall Partnership NHS Foundation Trust Bodmin United Kingdom; 4 Royal Cornwall Hospitals NHS Trust Truro United Kingdom; 5 UNEEG medical A/S Alleroed Denmark; 6 Department of Basic & Clinical Neuroscience King's College London London United Kingdom; 7 Harvard T.H. Chan School of Public Health Harvard University Boston, MA United States; 8 Department of Primary Care and Public Health School of Public Health Imperial College London London United Kingdom

**Keywords:** epilepsy, remote monitoring, electroencephalography, EEG, seizures, home care services, mental health

## Abstract

**Background:**

Electroencephalography (EEG) monitoring is a key tool in diagnosing and determining treatment for people with epilepsy; however, obtaining sufficient high-quality data can be a time-consuming, costly, and inconvenient process for patients and health care providers. Remote EEG monitoring has the potential to improve patient experience, data quality, and accessibility for people with intellectual or developmental disabilities.

**Objective:**

The purpose of this scoping review is to provide an overview of the current research evidence and knowledge gaps regarding the use of remote EEG monitoring interventions for adults with epilepsy.

**Methods:**

The PRISMA-ScR (Preferred Reporting Items for Systematic Reviews and Meta-Analyses extension for Scoping Reviews) and Population, Intervention, Comparator, Outcome, and Study (PICOS) frameworks will be used to structure the review. Searches will be conducted in 6 databases (PubMed, MEDLINE, Embase, CINAHL, Web of Science, and ClinicalTrials.gov) for articles published in English that evaluate at least one out-of-hospital EEG monitoring intervention or device for adults with epilepsy. A descriptive analysis will be conducted to summarize the results; key themes and gaps in the literature will be discussed.

**Results:**

Results will be included in the scoping review, which will be submitted for publication by April 2022.

**Conclusions:**

This scoping review will summarize the state of the field of remote EEG monitoring interventions for adults with epilepsy and provide an overview of the strengths, weaknesses, and gaps in the research.

**International Registered Report Identifier (IRRID):**

PRR1-10.2196/33812

## Introduction

### Background

Accurate measurement and detailed understanding of a person’s seizures are key elements in the diagnosis, classification, and treatment of epilepsy. The use of electroencephalography (EEG) for this purpose is well established [1]; however, routine EEG recordings often do not capture epileptiform activity or seizures as patients can have a low frequency of epileptic activity [2]. Long-term video-EEG monitoring is used to optimize treatment, but can cost thousands of dollars to conduct, requires patients to spend days in the hospital, and might not capture the semiology of everyday life seizures [3-5]. This is particularly a problem for patients with comorbidities, such as intellectual or developmental disabilities (IDD), for whom diagnosis can be more difficult and hospital-based monitoring intolerable [6]. This population remains underrepresented in research [7] and there is a lack of data on misdiagnosis relating to epilepsy in people with IDD [8]. This highlights a clear need for remote EEG monitoring systems, which have the potential to provide a less disruptive means of gathering objective seizure data, without relying on patient or observer reports of seizures.

### Rationale

A variety of monitors and alarms are available to support at-home monitoring of epilepsy and seizure detection [9] (Table 1) and some previous reviews have been conducted in this field [10-13]. One review found that the devices available on the market focused primarily on monitoring non-EEG signals [10]; however, studies of implantable devices were excluded from all of the reviews [10-13]. Three of the reviews also reported a need for further evidence of the clinical effectiveness and usability of the at-home seizure monitoring systems assessed but concluded that the systems did have potential to provide clinically useful data, be acceptable to patients, and empower patient self-monitoring and self-management [10-12]. However, none of these reviews provided an overview of remote EEG monitoring devices for adults with epilepsy and a search of PROSPERO (International Prospective Register of Systematic Reviews) using the terms (epilepsy AND remote EEG monitoring) did not find any reviews in progress on this topic. This demonstrates the need for a comprehensive overview of the different means of conducting remote EEG recordings that are being developed and evaluated for people with epilepsy.

No published or in progress reviews were identified that focused on adults with epilepsy and IDD. Given the potential value of remote EEG monitoring for all people with epilepsy, but particularly people with epilepsy and IDD, an overview of the devices being developed to deliver remote EEG monitoring is needed. This review will include studies evaluating remote monitoring interventions in any adults with epilepsy; however, effort will be made to identify studies in the population of adults with epilepsy and IDD, and they will be highlighted in the analysis. This scoping review will summarize the state of the field of all remote EEG monitoring interventions for adults with epilepsy, the strengths and weaknesses of the interventions and the studies evaluating them, and gaps in the literature. An overview of the current state of the literature and the gaps can be used to inform future directions for research and development.

**Table 1:** Types of seizure detection systems for at-home monitoring of epilepsy.

Detection system	Description	What it monitors
Wearable sensors [9]	Wearable device (such as a watch or other wrist-worn sensor)	Primarily movement and heart rate, some can also measure other skin properties (temperature, sweat, etc)
Apps/subscriptions [9]	Mobile app, usually linked with a wearable sensor	Can send alerts to people about a seizure, track location via GPS, track seizures, send medication reminders, etc
Bed monitors [9]	Sensors placed under a mattress, linked with a pager	Primarily movement and sound, some can also monitor vomit and urination
Video monitors [9,13]	Infrared camera device, linked with app, pager, or other monitoring tool	Primarily movement, but can also record audio and other visible signs
Ambulatory scalp electroencephalography [14-17]	Electrodes/sensors attached to scalp	Electrical brain activity
Subcutaneous electroencephalography [18,19]	Electrode implanted under skin, attached to small logging device	Electrical brain activity
Intracranial electroencephalography [20]	Electrode implanted in the brain, attached to small logging device	Electrical brain activity

### Aim and Research Questions

The aim of this scoping review is to identify and summarize the current state of the literature on remote EEG monitoring interventions for adults with epilepsy. This review will be based on the following research question: What interventions are being evaluated and delivered to enable out-of-hospital EEG monitoring of epileptic seizures in adults, particularly those with IDD?

## Methods

### Frameworks

The PRISMA-ScR (Preferred Reporting Items for Systematic Reviews and Meta-Analyses Extension for Scoping Reviews; Multimedia Appendix 1) [21] and Population, Intervention, Comparator, Outcome, and Study (PICOS) frameworks [22] were used to build the search strategy (Table 2) and provide a framework for the review.

**Table 2:** Population, Intervention, Comparator, Outcome, and Study (PICOS) framework.

	Description of inclusion criteria
Population	All adults (≥18 years old) with epilepsy will be included, but there will be a specific examination of adults with intellectual and developmental disabilities if possible
Intervention	Remote EEG^a^ monitoring interventions
Comparator	No comparator will be required
Outcome	The primary outcome will be the evidence for the remote monitoring technology’s ability to record EEG for subsequent detection of seizures. Secondary outcomes will include the different remote monitoring types and the strengths and weaknesses of the monitoring interventions and the studies.
Study types	All study types that evaluate a relevant intervention will be eligible for inclusion. Protocols, reviews, meta-analyses, and conference or poster abstracts where no full text is available will be excluded.

^a^EEG: electroencephalography.

### Search Strategy

This review will search 6 databases to identify potentially relevant references: PubMed, MEDLINE, Embase, CINAHL, Web of Science, and ClinicalTrials.gov. A preliminary review of the literature identified relevant Medical Subject Headings (MeSH) terms and keywords, which were grouped into three themes to structure the search (Table 3). They will be strung together in the following way when searching the databases: population (MeSH OR keywords) AND epilepsy (MeSH OR keywords) AND remote EEG monitoring (MeSH OR keywords). Multimedia Appendix 2 provides a sample search string and the number of results returned in PubMed and Web of Science.

**Table 3:** Search string.

Category	Medical Subject Headings (MeSH)	Keywords (in title or abstract)
Population	Adult OR Persons with Mental Disabilities OR Intellectual Disability	Adult OR adults OR “developmental disabilit*” OR “learning disabilit*” OR “intellectual disabilit*” OR “learning disorder*” OR “developmental disorder*” OR “special need*” OR “mental retardation” OR autis* OR “Down syndrome” OR “fetal alcohol”) NOT (child* OR pediatric OR paediatric OR adolescen* OR teen*)
Epilepsy	Epilepsy OR Seizures	Epilepsy OR seizure OR epileptic OR convulsion OR ictal OR preictal OR postictal OR interictal OR epileptiform
Remote electroencephalographic monitoring	Monitoring, Ambulatory OR Electrodes, Implanted OR Electroencephalography	((“Remote monitor*” OR implant* OR sensor* OR wearable* OR device* OR detection* OR alert* OR home OR mobile) AND (EEG OR electroencephalograph* OR seizure*)) OR “Long-term electroencephalographic monitoring” OR “continuous electroencephalographic monitoring” OR “continuous EEG” OR LTM OR “intracranial EEG” OR “intracranial electroencephalography” OR iEEG OR ((ambulatory OR subcutaneous OR subscalp OR subgaleal OR subdermal OR epicranial OR epiosteal OR “scalp-based” OR “behind the ear” OR “behind-the-ear”) AND (EEG OR electroencephalography))

### Inclusion Criteria

All adults (≥18 years old) with epilepsy will be included to ensure that there is good coverage of the literature, but studies with participants with IDD and epilepsy will be identified and analyzed independently as well. Interventions will be included given that they support at-home EEG monitoring of epileptic seizures; this can be as a wearable device or an implant. No comparator is required and all study types will be eligible for inclusion, given that they are evaluating such an intervention (at any stage).

### Exclusion Criteria

Any studies focusing on pediatric populations or evaluating remote monitoring interventions for epilepsy that do not use EEG (including electronic seizure diaries, motion sensors, and video monitors) will be excluded. Studies that do not evaluate the intervention (such as protocols, reviews, and abstracts without full texts available) and any duplicates will also be excluded. Studies that are not published in English after 2011 will not be eligible for inclusion.

### Screening and Article Selection

The references will be stored, and duplicates removed, using the citation management software EndNote X9 (Clarivate). The EndNote X9 search function will also be used to conduct an initial screening of the references based on keywords from the search strategy. The included studies in any relevant reviews identified in the screening will be hand searched to make sure that no studies fitting the inclusion criteria were missed in the original search. If any relevant studies are identified, they will be added to the list for full-text review. The titles and abstracts will be screened, and a full-text review conducted, by one of the authors (MMI) to determine final eligibility. A second reviewer will independently validate the title and abstract screening and full-text selection.

### Data Extraction

Two reviewers will extract data from the included studies into a predeveloped form (Table 4).

**Table 4:** Article information and data extraction.

Article information and data to be extracted	
**General study information**	
	Year of publication
	Sample size
	Study type
	Target population (if specified, eg, those with an intellectual or developmental disability)
**Intervention**	
	Type of intervention
	Description of intervention features/components
	Degree of free movement when using (static or mobile)
	Duration of patient use
**Evaluation**	
	Main findings regarding seizure detection (eg, sensitivity, specificity, false-alarm rate, safety, percentage of seizures captured, success at answering clinical question)
	Acceptability/patient perceptions
	Benefits of the remote electroencephalographic monitoring intervention
	Limitations of the remote electroencephalographic monitoring intervention
	Strengths and weaknesses of the study

### Data Analysis and Synthesis

The primary aim of this scoping review is to provide an overview of the state of the literature, so the analysis will focus on describing the research being conducted, the strengths and weaknesses of the included studies, and key implications and considerations for future research. Specific analyses relating to the interventions will depend on the types of data collected by the included studies. For example, thematic analysis will be conducted to provide an overview of qualitative data relating to acceptability (ie, patient experiences, clinical acceptability, concerns) and quantitative data about seizure detection will be summarized by providing a breakdown of main findings by the type of intervention.

The analysis will also identify any studies that include or focus on patients with IDD. These studies will be examined separately to identify any unique challenges, considerations, or impacts of the remote EEG monitoring interventions in this population. The data will be analyzed in the same way as the general analysis to enable comparison between population groups, enabling any potential differences between patients with IDD and patients without to be identified. This could include differences in study outcomes (findings relating to seizure detection, acceptability, and patient perceptions) as well as study designs, strengths, and weaknesses. The purpose of this exploratory analysis is to identify areas for further investigation and to inform the design of future studies of adults with epilepsy and IDD.

## Results

The study is expected to begin in February 2022 and be completed in April 2022.

## Discussion

This scoping review will provide an overview of the state of the literature regarding clinical and research data on remote EEG monitoring interventions for adults with epilepsy. This section will use the data extracted from the studies to explore what conclusions can be drawn, the limitations of the scoping review, and key areas for future research. A special focus will be placed on people with epilepsy and IDD, and studies investigating interventions in this population will be summarized and discussed in a subsection. The summary of current interventions, and the strengths and weaknesses of those interventions and the studies evaluating them, will help to inform the development of new remote EEG monitoring strategies and improve the quality of their evaluation.

## Appendix

Multimedia Appendix 1 PRISMA-ScR checklist.

Multimedia Appendix 2 Sample search strings.

## Abbreviations

EEG: electroencephalography
IDD: intellectual or developmental disabilities
MeSH: Medical Subject Headings
PRISMA-ScR: Preferred Reporting Items for Systematic Reviews and Meta-Analyses Extension for Scoping Reviews
PROSPERO: International Prospective Register of Systematic Reviews
