# Supplementary material for: Remote Electroencephalography Monitoring of Epilepsy in Adults: Protocol for a Scoping Review
Source: JMIR Res Protoc. 2022 Feb 25;11(2):e33812. doi: 10.2196/33812 (PMC8917432; doi:10.2196/33812)
Supplement: Multimedia Appendix 2 [file resprot_v11i2e33812_app2.docx]

### Appendix B. Sample Search Strings

| **Database** | **Search string** | **Results** |
| --- | --- | --- |
| PubMed | ((Adult[MeSH Terms] OR Persons with Mental Disabilities[MeSH Terms] OR Intellectual Disability[MeSH Terms]) OR (Adult[Title/Abstract] OR adults[Title/Abstract] OR "developmental disabilit*"[Title/Abstract] OR "learning disabilit*"[Title/Abstract] OR "intellectual disabilit*"[Title/Abstract] OR "learning disorder*"[Title/Abstract] OR "developmental disorder*"[Title/Abstract] OR "special need*"[Title/Abstract] OR "mental retardation"[Title/Abstract] OR autis*[Title/Abstract] OR "Down syndrome"[Title/Abstract] OR "fetal alcohol"[Title/Abstract]) NOT (child*[Title/Abstract] OR pediatric[Title/Abstract] OR paediatric[Title/Abstract] OR adolescen*[Title/Abstract] OR teen*[Title/Abstract])) AND ((Epilepsy[MeSH Terms] OR Seizures[MeSH Terms]) OR (Epilepsy[Title/Abstract] OR seizure[Title/Abstract] OR epileptic[Title/Abstract] OR convulsion[Title/Abstract] OR ictal[Title/Abstract] OR preictal[Title/Abstract] OR postictal[Title/Abstract] OR interictal[Title/Abstract] OR epileptiform[Title/Abstract])) AND ((Monitoring, Ambulatory[MeSH Terms] OR Electrodes, Implanted[MeSH Terms] OR Electroencephalography[MeSH Terms]) OR ((("Remote monitor*"[Title/Abstract] OR implant*[Title/Abstract] OR sensor*[Title/Abstract] OR wearable*[Title/Abstract] OR device*[Title/Abstract] OR detection*[Title/Abstract] OR alert*[Title/Abstract] OR home[Title/Abstract] OR mobile[Title/Abstract]) AND (EEG[Title/Abstract] OR electroencephalograph*[Title/Abstract] OR seizure*[Title/Abstract])) OR "Long-term electroencephalographic monitoring"[Title/Abstract] OR LTM[Title/Abstract] OR "continuous electroencephalographic monitoring"[Title/Abstract] OR "continuous EEG"[Title/Abstract] OR "intracranial EEG"[Title/Abstract] OR "intracranial electroencephalography"[Title/Abstract] OR iEEG[Title/Abstract] OR "ambulatory EEG"[Title/Abstract] OR "ambulatory electroencephalography"[Title/Abstract] OR "subcutaneous EEG"[Title/Abstract] OR "subcutaneous electroencephalography"[Title/Abstract] OR "subscalp EEG"[Title/Abstract] OR "subscalp electroencephalography"[Title/Abstract] OR "subgaleal EEG"[Title/Abstract] OR "subgaleal electroencephalography"[Title/Abstract] OR "subdermal electroencephalography"[Title/Abstract] OR "subdermal EEG"[Title/Abstract] OR "epicranial EEG"[Title/Abstract] OR "epicranial electroencephalography"[Title/Abstract] OR "epiosteal EEG"[Title/Abstract] OR "epiosteal electroencephalography"[Title/Abstract] OR "scalp-based EEG"[Title/Abstract] OR "scalp-based electroencephalography"[Title/Abstract] OR "behind the ear EEG"[Title/Abstract] OR "behind the ear electroencephalography"[Title/Abstract])) | 20,120 |
| Web of Science Core Collection ^a,b^ | ((Adult OR "Persons with Mental Disabilities" OR adults OR "developmental disabilit*" OR "learning disabilit*" OR "intellectual disabilit*" OR "learning disorder*" OR "developmental disorder*" OR "special need*" OR "mental retardation" OR autis* OR "Down syndrome" OR "fetal alcohol") NOT (child* OR pediatric OR paediatric OR adolescen* OR teen*)) AND (Epilepsy OR seizure OR epileptic OR convulsion OR ictal OR preictal OR postictal OR interictal OR epileptiform) AND ((monitoring NEAR/2 ambulatory) OR (electrode NEAR/2 implant*) OR (("Remote monitor*" OR implant* OR sensor* OR wearable* OR device OR detection OR alert) AND (EEG OR electroencephalograph* OR seizure)) OR "Long-term electroencephalographic monitoring" OR LTM OR "continuous electroencephalographic monitoring" OR "continuous EEG"OR "intracranial EEG"OR "intracranial electroencephalography" OR iEEG OR "ambulatory EEG" OR "ambulatory electroencephalography" OR "subcutaneous EEG" OR "subcutaneous electroencephalography" OR "subscalp EEG" OR "subscalp electroencephalography" OR "subgaleal EEG" OR "subgaleal electroencephalography" OR "subdermal electroencephalography" OR "subdermal EEG" OR "epicranial EEG" OR "epicranial electroencephalography" OR "epiosteal EEG" OR "epiosteal electroencephalography" OR "scalp-based EEG" OR "scalp-based electroencephalography" OR "behind the ear EEG" OR "behind the ear electroencephalography") | 1,325 |

^a^Web of Science does not use MeSH terms, so all MeSH terms have been included as keywords.

^b^Keywords searched in ‘Topic,’ which includes title, abstract, author keywords, and Keywords Plus.
